# Variability in competitive decision-making speed and quality against exploiting and exploitative opponents

**DOI:** 10.1038/s41598-021-82269-2

**Published:** 2021-02-03

**Authors:** Benjamin James Dyson

**Affiliations:** 1 Department of Psychology, University of Alberta, P-217 Biological Sciences Building, Edmonton, AB T6G 2E9 Canada; 2 Ryerson University, Toronto, Canada; 3 University of Sussex, Brighton, UK

**Keywords:** Psychology, Human behaviour

## Abstract

A presumption in previous work has been that sub-optimality in competitive performance following loss is the result of a reduction in decision-making time (i.e., post-error speeding). The main goal of this paper is to test the relationship between decision-making speed and quality, with the hypothesis that slowing down decision-making should increase the likelihood of successful performance in cases where a model of opponent domination can be implemented. Across Experiments 1–3, the speed and quality of competitive decision-making was examined in a zero-sum game as a function of the nature of the opponent (unexploitable, exploiting, exploitable). Performance was also examined against the nature of a credit (or token) system used as a within-experimental manipulation (no credit, fixed credit, variable credit). To compliment reaction time variation as a function of outcome, both the fixed credit and variable credit conditions were designed to slow down decision-making, relative to a no credit condition where the game could be played in quick succession and without interruption. The data confirmed that (a) self-imposed reductions in processing time following losses (post-error speeding) were causal factors in determining poorer-quality behaviour, (b) the expression of lose-shift was less flexible than the expression of win-stay, and, (c) the use of a variable credit system may enhance the perceived control participants have against exploitable opponents. Future work should seek to disentangle temporal delay and response interruption as determinants of decision-making quality against numerous styles of opponency.

## Introduction

How we choose to approach, engage and ultimately remove ourselves from competitive environments is a critical component in understanding industrial, political, educational and gambling behaviour. In the context of gambling, research has highlighted how problematic gambling behavior can be characterized as cyclic in nature (e.g.^1,2^), with cognitive psychology providing insights into how these cycles appear. Two empirical observations are key. First, the quality of decision-making following negative outcomes (e.g., losses) tends to be sub-optimal relative to the decision-making following positive outcomes (e.g.^3^). Second, an assumed cause of the deterioration of decision-making following negative outcomes is a reduction in processing time allocated to actions following losses (e.g.^4^). This *post-error speeding* is manifest in competitive environments in which an opponent behaves in a random way^5,6^, characteristic of many real-world devices such as fixed-odds betting terminals (FOBTs^7^). *Post-error speeding* also represents the flip side of *post-reinforcement pauses*^8^. Here, scaled temporal delays following wins may be initiated by the device itself (such longer audio feedback when the pay-out is larger^9^), or, by the player themselves who simply wishes to revel in the positive state that winning affords.

However, an important determinant of *post-error speeding* remains is the interaction between the nature of the opponent and individual performance. Broadly, three classes of opponent can be identified. First, the opponent may play in an *unexploitable* way, usually taking the form of equal but random distribution of responses defined by the finite length of the game series. For example, in the context of the game Matching Pennies the opponent would select 50% heads and 50% tails, or, in the context of the game Rock, Paper, Scissors the opponent would select each of the three responses 33.3% of the time. This type of random play is the only way to guarantee the absence of loss maximization (but neither does it provide win maximization). Second, playing against *exploitable* opponents allow participants to maximize their wins. Here, opponent responding will be predictable (such as over-playing one response over another; item biases) and if this predictability is utilized by the player their win rate will increase. Third, *exploiting* opponent provide the threat of loss maximization. Here, opponents may examine participant response distributions for similar item biases and respond in a manner that would maximize losses for the player. Previous data suggests that *post-error speeding* is most likely to arise either when there is interaction with an *unexploitable* opponent (see above), or, when there is interaction with an *exploitable* opponent but the individual fails to acquire the appropriate mental model for domination^10^. In contrast, *post-error slowing* is most likely during successful exploitation, with the degree of slowing predicted by the degree of exploitation^10^ and as errors become rare events^11^. Thus, the main goal of this paper is to specifically test the relationship between decision-making speed and quality, with the hypothesis that slowing down decision-making following losses increases the likelihood of future successful performance in cases where a successful model of opponent domination can be implemented.

The view that there is a link between negative outcomes and poorer-quality (faster) decision-making would appear to contrast other work, in which loss orients attention to the failed action or stimulus (for examples in the context of arm wrestling and Rock, Paper, Scissors; see^12^). Specifically^13^, argue that the phasic increase in arousal driven by negative outcomes also increases attention towards task-relevant features. While one might anticipate that increasing attentional resource might enhance the quality of subsequent decision-making under certain contexts (e.g., expected value maximization^14^), Yechiam and Hochman^13^ also report the tendency for losses to lead to an increased likelihood of exploration (*switch*) behaviour (see also^15,16^). Furthermore, persistent effects of continued loss lead to ‘restlessness’ whereas continued wins lead to ‘calmness,’ essentially the expression of *lose-shift* and *win-stay* behaviour in the long run and even across tasks^17^. Therefore, the increased investment of attention does not necessarily translate to better performance. Specifically, in the context of competition any natural predictability in behaviour such as *lose-shift* runs the risk of exploitation.

In addition to the self-imposed speeding up or slowing down of decision-making as a function of outcome, further variability in the speed of play is apparent in the use of a ‘credit’ (or ‘token’) systems. Such systems are apparent in a variety of real-world devices such as gumball, arcade and slot machines. The gumball device is an example of a *fixed credit* system where the gumball desirer has to provide the machine with one (and only one) coin in order to receive one gumball: the coin puts the individual ‘in credit’ for one gumball, but to receive a second gumball the process must be completely repeated. This may be contrasted with a *variable credit* system represented by arcade and slot machines. Here, players must again provide coins to accrue credits but the system allows for more than one credit to be stored. For example, if our individual has given an arcade machine two quarters (25c), after losing the first game, the player can instantaneously begin the second game without pausing to insert more coins. Both the *fixed credit* and *variable credit* systems can, of course, be contrasted with *no credit* systems where the system can be played multiple times without interruption. These different types of a credit system create clear parallels with the allocation of time and the quality of decision-making described above. Specifically, a device that only accepts one credit at a time (i.e., *fixed credit*) creates a naturally slow cycle of play relative to the absence of such a system (i.e. *no credit*). The intermediate scheme (i.e., *variable credit*) allows for credit to be stored, giving the player the opportunity to decide when and how many credits to input, allowing for the possibility of multiple plays in quick succession. Under this reading, *variable credit* systems then appear to be a rather cynical design feature of gambling devices, given the joint observations that quicker processing times are more likely to lead to sub-optimal decision-making^5^ and that pathological gamblers prefer faster playing machines^9^. The use of *variable credit* systems as promoting poorer rather than better quality decision-making is further reinforced by the literature on illusion of control^18,19^. Here, offering *any* kind of choice to individuals increases their perception of control in random environments (e.g., allowing individuals to select their own lottery numbers^20^). While illusion of control is less likely to persist across multiple interactions^21^, observations such as the *hot hand fallacy* and *gambler’s fallacy*^22,23^ continue to represent erroneous beliefs about the predictability of future events over the long run.

Under the assumption that subsequent decision-making following negative outcomes tends to be faster and sub-optimal, establishing exactly what represents ‘sub-optimal’ decision-making is determined by the specific competitive context and the degree to which outcomes are a function of skill and/or luck^24^. One natural place to start is by examining behaviour when participants interact with random (i.e., *unexploitable*) agents in competitive zero-sum games. These types of game can be used to measure the degree of deviation from optimal decision-making since ideal response distributions can be clearly set out, opponent characteristics can be perfectly controlled, the resolution of competition is fast yielding a high signal-to-noise ratio, and, games are both intuitive and often fun to play thereby providing participants with intrinsic motivation in a laboratory setting (see^25,26^, for a discussion of some of these issues).

Competitive environments are characterized by the mutual goals of maximizing wins and minimizing losses^27^. The only way to minimize losses in zero-sum games is to behave in according with a mixed-strategy (MS^28–30^; see also *minimax solution*^31^). Here, all actions must be randomized and the selection of the next action must not be contingent on the outcome of the previous action. Unfortunately, such behaviour runs counter to reinforcement learning heuristics. According to the keystone principles of operant conditioning (e.g.^32^), we will be more likely to repeat an action in the light of reinforcement (*win-stay*) and more likely to change an action in the light of punishment (*lose-shift*). Despite the historic view that the mechanisms associated with punishment and reward are simply the inverse of one another, there is converging evidence from a number of fields to suggest that they exist and operate independently of one another^33^. In particular, *lose-shift* appears to be a more robust phenomenon than *win-stay,* possibly in part due to the high cost of ‘losing’ from an evolutionary perspective^34,35^. Fundamental differences between reinforcement learning principles are further supported by animal work in which *lose-shift* mechanisms are also anatomically distinct from *win-stay* mechanisms (c.f., lesioning of the ventrolateral striatum), where *lose-shift* represents a “choice reflex” within the animal brain^36^, p. 1^37^. Differences in the degree to which *win-stay* and *lose-shift* behaviour are under cognitive control are similarly reflected in human work in which responses following wins tend to approach MS whereas responses following losses reveal a higher-than-expected level of shift behavior^3,6^. Furthermore, manipulating the value of wins modulates the degree of *win-stay* behavior whereas manipulating the value of losses does not change the degree of *lose-shift* behavior^5,35^. Once again, the main message here though is that while such reinforcement learning principles are contingent on both environment and species^38–40^, natural predictability in behaviour expressed via *win-stay* and/or *lose-shift* runs the risk of exploitation in competitive environments.

## Experiment 1

As an initial test of competitive decision-making in Experiment 1, participants interacted with a computer opponent playing according to a mixed-strategy (MS) in the zero-sum game of Rock, Paper, Scissors (RPS; see^41^ for a review). In terms of defining optimal and sub-optimal performance, the Nash^42^ equilibrium for RPS against an opponent playing mixed-strategy is for the participant to also play mixed-strategy. In this respect, the *no credit* condition served as an attempted replication of the data from^3,5^ (baseline), and^6^, where each trial consisted of a single response only. Here, performance should approximate optimal MS following *wins* where the single stay response and the two shift responses are played roughly 33.3% of the time. Conversely, performance after negative outcomes (both losses and draws) should be characterized by an increase in shift behaviour over the 66.6% predicted by optimal performance. Given the *unexploitable* nature of the opponent, performance should also be characterized by post-error speeding^5,10,43^.

In Experiment 1, variations in a credit system were used to establish different temporal delay conditions (see Supplementary Materials A and B). In the *no credit* condition, participants simply made a single response selection on each of the 90 trials. For the *fixed credit* condition, participants had to ‘insert one credit’ on each of the 90 trials before they could make their game decision (c.f.^43,44^). This condition should slow down the cycle of play by providing mandatory response interruptions (and hence, regular temporal delays). If slowing down decision-making time increases the quality of decision-making, then there should be a reduction in shift behaviour exhibited following negative outcomes^45^. In the *variable credit* condition, participants had the same 90 credits in the *fixed credit* condition, but when and how many credits to insert was the participant’s decision. The same constraint existed in that participants could not play the trial unless they had at least 1 credit stored on the computer. Thus, the *variable credit* condition should also slow down the cycle of play by providing voluntary response interruptions (and hence, intermittent temporal delays). Since multiple credits could be inserted at any point during the condition, the degree of interruption should be intermediate, somewhere between the *no credit* and *fixed credit* condition. Therefore, the reduction in shift behaviour following negative outcomes should be more than that shown in the *fixed credit* condition, but less than that shown in the *no credit* condition. Finally, if pausing serves as way to maintain better rather than worse quality decision-making then participants should input more credits following positive relative to negative outcomes. All manipulations of trial lag expressed via variations in the credit system were within-participants to reduce the noise traditionally associated with between-participant designs (e.g.^46–48^).

### Method

#### Participants

36 individuals were analysed in the study: 25 were female, 3 were left-handed, with mean age = 20.11 (*sd* = 3.27). One individual was replaced due the recording of only 89 credits in the *variable credit* condition, and a second individual was replaced as a result of playing Scissors 100% and 99% of the time during the *fixed* and *no credit* conditions. Replaced participants undertook the experiment using the same counterbalanced order as the removed participants. All studies reported in this paper were approved by Research Ethics Board 2 at the University of Alberta under the protocol PRO00086116. All experiments were performed in accordance with relevant guidelines and regulations, including obtaining written informed consent. All participants completed the studies for course credit and no participant took part in multiple experiments.

#### Stimuli and apparatus

Pictures of two gloved hands representing the 9 interactions between participant and opponent during Rock, Paper, Scissors were used from^5^ (approximate on-screen size 10.5 cm × 4 cm). Stimulus presentation and response monitoring was conducted by Presentation (version 18.3, build 07.18.16).

#### Design

Participants completed 270 round of RPS split across three counter-balanced blocks of 90 trials each. In the *no credit* condition, participants made one response per round involving the selection of Rock, Paper or Scissors. In the *fixed credit* condition, participants had to make two responses per round: a response to insert one credit and a second response that allowed them to pick their response for that trial. In the *variable credit* condition, participants were allocated 90 credits at the start of the block, inserted as many as credits as desired, but could only play a round if their current credit score was 1 or above. For both *fixed* and *variable* conditions, if the number of inserted credits fell below 1, a warning sign appeared on screen and participants could not proceed with game responses. All opponents played 30 Rock, 30 Paper and 30 Scissors responses in a randomized order across each block (i.e., *unexploitable*). In this and all subsequent experiments, credit manipulation was a within-participants factor and opponency was a between-participants factor split across Experiments (1 = *unexploitable*, 2 = *exploiting*, 3 = *exploitable*).

#### Procedure

On-screen instructions from the various conditions are presented in Supplementary Information A and examples of the on-screen displays are presented in Supplementary Information B. At each trial and for each block, the participant’s current score was displayed for 500 ms, with a credit counter starting at 90. In the *no credit* condition, participants simply had to select 4, 5, or 6 on the number pad corresponding to the selection of RPS to decrease the counter by 1. In the *fixed credit* condition, a current credit counter was also displayed and would be red when the current number of credits was 0. Participants were always prompted with the display of ‘Insert 1 Credit’ at every trial and had to press 0 on the number pad before selecting their choice of RPS. The *variable credit* condition was identical to the fixed credit condition, apart from the prompt of ‘Insert × Credits’ at every trial. Here, participants could simply select RPS if their current credit count was above 0 (after which 1 was then subtracted from their current count), or before then, could press 0 on the number pad to transfer credits to the current credit counter to a maximum of 90 credits at any point(s) during the block.

Following the selection of the game response, RPS selections were displayed for opponent (on the left; blue glove) and participant (on the right; white glove) for 1000 ms. This display was removed for 500 ms and then the outcome of the trial was presented for 1000 ms in the form of ‘WIN’ (+ 1; green font), ‘LOSS’ (− 1; red font), or ‘DRAW’ (0; yellow font) as appropriate. The outcome was removed and the player’s score was updated across a 500 ms period, after which the next trial began with the prompt appropriate for that condition. After the completion of all three conditions, participants were thanked for their time and debriefed.

### Results

#### Item and outcome biases

Item and outcome biases were initially analysed using a one-way repeated measures ANOVA. The proportion of Rock selection did not significantly vary as a function of *no*, *variable* and *fixed* credit conditions [F(2,70) = 1.54, MSE = 0.004, *p* = 0.222, ƞ_p_^2^ = 0.042] nor did the proportion of Rock selection significantly differ from the expected value of 33.3% as assessed by a one-sampled t-test (e.g.^26,49^ Rapoport and Budescu, 1992; Hochman and Yechiam, 2011): *t*[35] = 1.79, p = 0.082. Similarly, the proportion of wins did not significantly vary as a function of credit conditions [F(2,70) = 0.62, MSE = 0.002, *p* = 0.543, ƞ_p_^2^ = 0.017] nor did win outcomes significantly differ from the expected value of 33.3% as assessed by a one-sampled t-test: *t*[35] =  − 0.86, p = 0.397. The rough equivalency of wins (32.93%), losses (33.89%) and draws (33.18%) would be anticipated on the basis of an opponent playing MS in all conditions (see Table 1).

**Table 1:** Distribution of items and outcomes as a function of credit (no, variable, fixed) across Experiments 1–3.

	Experiment 1 (unexploitable)			Experiment 2 (exploiting)			Experiment 3 (exploitable)		
	Rock	Paper	Scissors	Rock	Paper	Scissors	Rock	Paper	Scissors
**Item biases**									
No	0.362 (0.014)	0.328 (0.010)	0.310 (0.013)	0.348 (0.008)	0.339 (0.009)	0.313 (0.007)	0.355 (0.020)	0.340 (0.020)	0.306 (0.018)
Variable	0.356 (0.012)	0.338 (0.014)	0.305 (0.013)	0.343 (0.010)	0.342 (0.011)	0.315 (0.008)	0.336 (0.020)	0.339 (0.021)	0.326 (0.020)
Fixed	0.337 (0.013)	0.360 (0.013)	0.303 (0.013)	0.333 (0.010)	0.349 (0.009)	0.319 (0.007)	0.356 (0.026)	0.307 (0.020)	0.337 (0.028)

	Win	Lose	Draw	Win	Lose	Draw	Win	Lose	Draw
**Outcome distribution**									
No	0.331 (0.008)	0.344 (0.008)	0.324 (0.009)	0.331 (0.009)	0.340 (0.010)	0.329 (0.009)	0.351 (0.010)	0.323 (0.007)	0.326 (0.010)
Variable	0.322 (0.008)	0.342 (0.007)	0.336 (0.008)	0.334 (0.007)	0.327 (0.008)	0.339 (0.007)	0.373 (0.007)	0.318 (0.007)	0.309 (0.005)
Fixed	0.334 (0.008)	0.331 (0.007)	0.335 (0.008)	0.313 (0.007)	0.335 (0.008)	0.352 (0.008)	0.335 (0.011)	0.328 (0.010)	0.337 (0.009)

Standard error in parenthesis.

The three possible items and three possible outcomes were also directly compared using arc-sine transformed data at the request of a reviewer, yielding equivalent conclusions. For Experiment 1, analyses of the arc-sine transformed proportion of *Rock, Paper, Scissor* responses as a function of *no*, *variable* and *fixed* credit conditions produced a significant main effect of item: [F(2,70) = 3.60, MSE = 0.020, *p* = 0.033, ƞ_p_^2^ = 0.093], in the absence of a significant interaction: [F(4,140) = 1.73, MSE = 0.005, *p* = 0.147, ƞ_p_^2^ = 0.047]. The slight over-representation of *Rock* (35.18%) relative to *Scissors* (30.61%; Tukey’s HSD, *p* < 0.05; but not *Paper*, 34.22%) was consistent with previous data (see Table 1). Analyses of the similarly arc-sine transformed proportion of *win*, *lose* and *draw* outcomes as a function of *no*, *variable* and *fixed* credit conditions did not produce a significant main effect of outcome: [F(2,70) = 0.86, MSE = 0.003, *p* = 0.426, ƞ_p_^2^ = 0.024], nor a significant interaction: [F(4,140) = 0.66, MSE = 0.004, *p* = 0.623, ƞ_p_^2^ = 0.018].

#### Reinforcement learning biases

**Figure 1:**
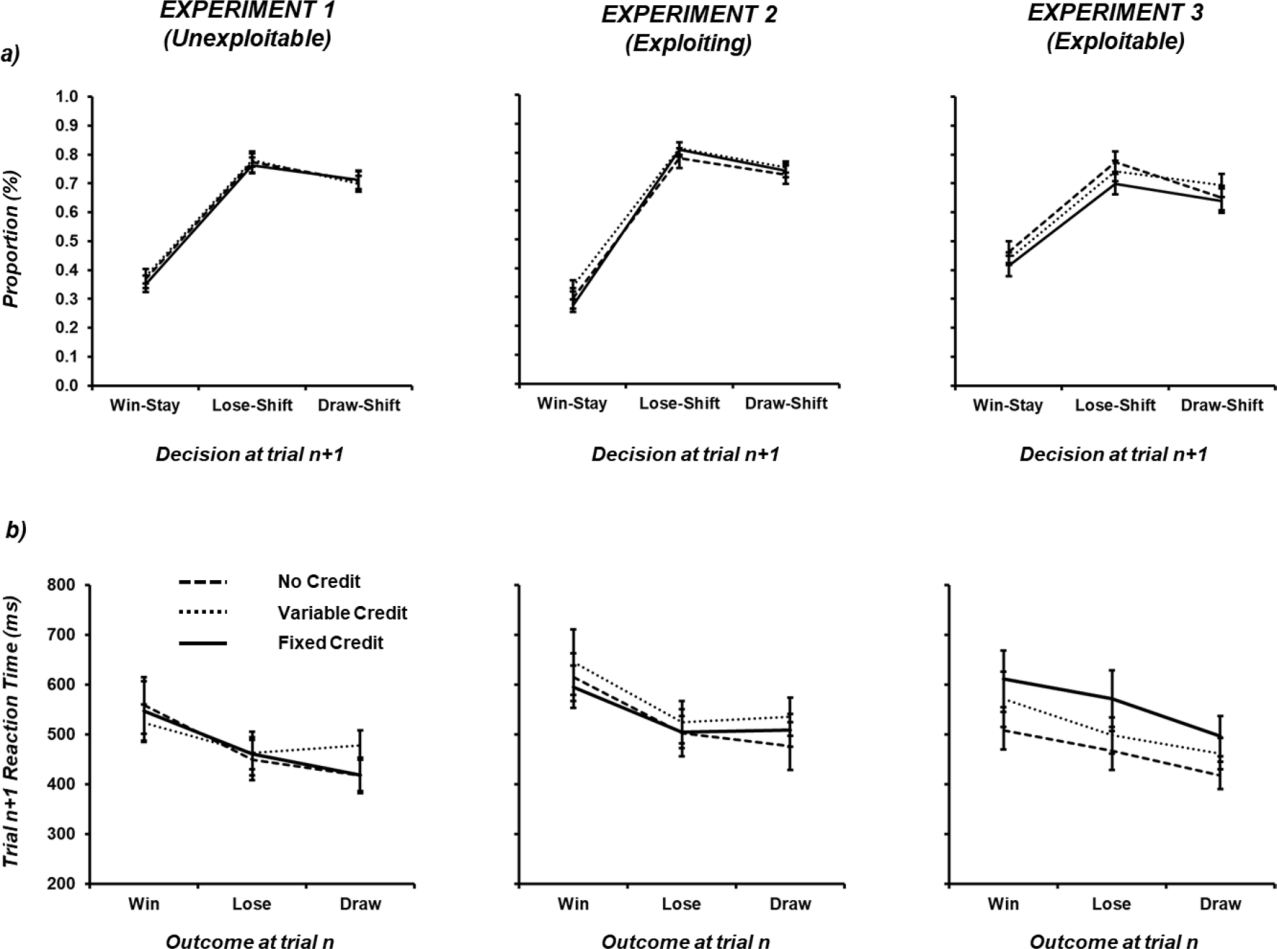
(**a**) Graph showing proportion of reinforcement learning biases (win-stay, lose-shift, draw-shift) under conditions of no, variable and fixed credit across three different opponent styles (Experiment 1 = *unexploitable*, Experiment 2 = *exploiting*, Experiments 3 = *exploitable*). (**b**) Graph showing reaction times at trial n + 1 separated by outcome at trial n (win, lose, draw) under conditions of no, variable and fixed credit across three different opponent styles. In both (**a**,**b**) error bars indicate ± 1 standard error.

Table 2 provides summary statistics for the three strategies at trial *n* + 1 as a function of trial *n* outcome. To assess traditional reinforcement learning biases, the proportion of *win-stay*, *lose-shift* and *draw-shift* were analysed as a function of condition using separate one-way repeated-measures ANOVAs, and, with respect to the value expected on the basis of MS behaviour (33.3% stay responses, 66.6% shift responses; see Fig. 1a) using one-sampled t-tests. Group average data are shown in Fig. 1a and individual data are shown in Supplementary Fig. C1. *Win-stay* behaviour did not alter as a function of condition: [F(2,70) = 0.46, MSE = 0.014, *p* = 0.632, ƞ_p_^2^ = 0.013] and the observed average value of 36.67% did not differ significantly from the expected value of 33.33% (*t*[35] = 1.10, *p* = 0.280). *Lose-shift* behaviour did not alter as a function of condition: [F(2,70) = 0.43, MSE = 0.007, *p* = 0.655, ƞ_p_^2^ = 0.012] but the observed average value of 77.23% did differ significantly from the expected value of 66.66% (*t*[35] = 4.75, *p* < 0.001). *Draw-shift* behaviour did not alter as a function of condition: [F(2,70) = 0.16, MSE = 0.011, *p* = 0.853, ƞ_p_^2^ = 0.005] and the observed average value of 70.65% did not differ significantly from the expected value of 66.66% (*t*[35] = 1.53, *p* = 0.136). The degree of shift behaviour following loss was greater than that following draw (*t*[35] = 2.91, *p* = 0.006; two-tailed).

**Table 2:** Proportion of strategy at trial *n* + 1 (*sta*y, *upgrade* [shift], *downgrad*e [shift]) relative to outcome at trial *n* (win, lose, draw) as a function of credit (no, variable, fixed) across Experiments 1–3.

	Experiment 1 (unexploitable)								
	No credit			Variable credit			Fixed credit		
	Win	Lose	Draw	Win	Lose	Draw	Win	Lose	Draw
Stay	0.376 (0.031)	0.234 (0.025)	0.294 (0.027)	0.384 (0.033)	0.220 (0.021)	0.302 (0.027)	0.357 (0.034)	0.246 (0.025)	0.294 (0.029)
Upgrade	0.329 (0.022)	0.385 (0.022)	0.380 (0.023)	0.316 (0.025)	0.390 (0.019)	0.372 (0.021)	0.345 (0.024)	0.367 (0.023)	0.346 (0.019)
Downgrade	0.295 (0.018)	0.382 (0.021)	0.327 (0.017)	0.300 (0.021)	0.390 (0.023)	0.326 (0.021)	0.298 (0.023)	0.388 (0.022)	0.360 (0.020)

	Experiment 2 (exploiting)								
	No credit			Variable credit			Fixed credit		
	Win	Lose	Draw	Win	Lose	Draw	Win	Lose	Draw
Stay	0.295 (0.035)	0.220 (0.020)	0.276 (0.021)	0.338 (0.035)	0.186 (0.021)	0.251 (0.025)	0.271 (0.033)	0.189 (0.020)	0.262 (0.024)
Upgrade	0.337 (0.024)	0.379 (0.018)	0.326 (0.019)	0.341 (0.025)	0.382 (0.021)	0.384 (0.018)	0.364 (0.026)	0.383 (0.022)	0.362 (0.028)
Downgrade	0.368 (0.023)	0.401 (0.021)	0.398 (0.020)	0.321 (0.024)	0.433 (0.019)	0.366 (0.024)	0.365 (0.023)	0.428 (0.025)	0.376 (0.021)

	Experiment 3 (exploitable)								
	No credit			Variable credit			Fixed credit		
	Win	Lose	Draw	Win	Lose	Draw	Win	Lose	Draw
Stay	0.463 (0.037)	0.225 (0.021)	0.350 (0.036)	0.439 (0.036)	0.257 (0.035)	0.307 (0.035)	0.415 (0.043)	0.302 (0.039)	0.360 (0.042)
Upgrade	0.288 (0.025)	0.370 (0.018)	0.363 (0.023)	0.285 (0.027)	0.343 (0.024)	0.365 (0.026)	0.292 (0.025)	0.316 (0.024)	0.325 (0.025)
Downgrade	0.250 (0.022)	0.405 (0.022)	0.286 (0.023)	0.376 (0.029)	0.400 (0.030)	0.327 (0.026)	0.293 (0.032)	0.382 (0.029)	0.315 (0.028)

Standard error in parenthesis.

#### Reaction times

**Figure 2:**
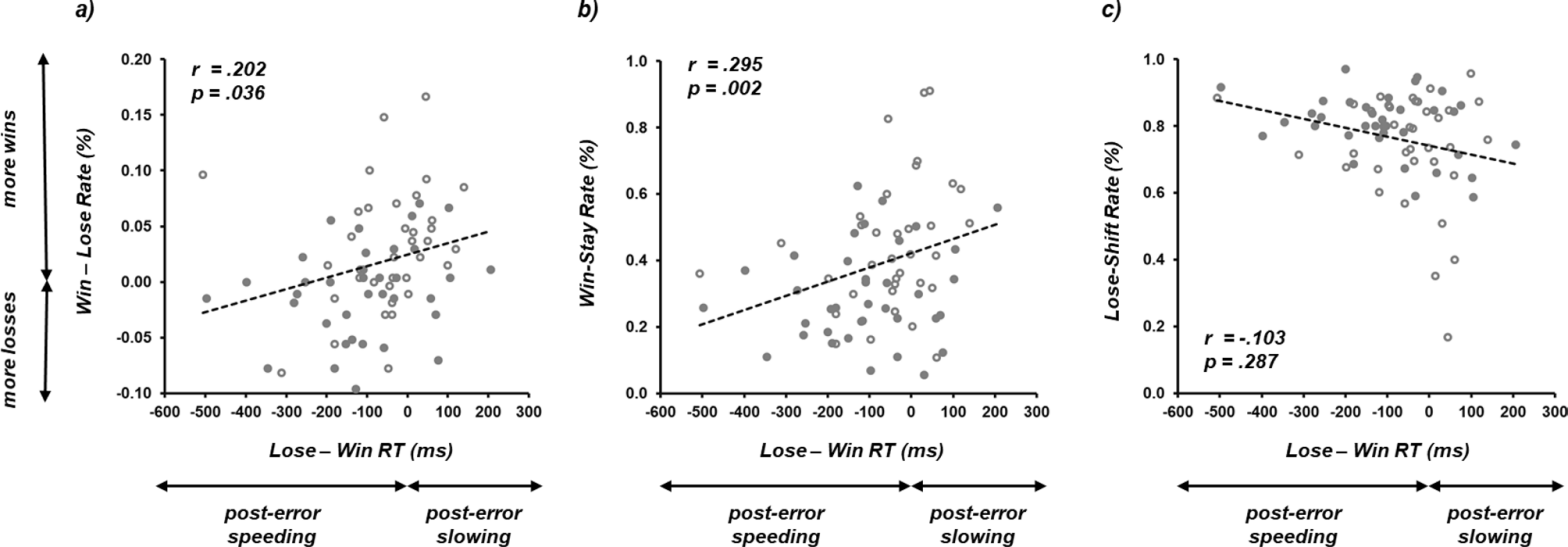
(**a**) Scatterplot comparing average lose minus win reaction time (RT) indexing the degree of *post-error speeding* (lose < win) or *post-error slowing* (lose > win) against the rate of wins minus losses indexing participants who were successful (more wins) or unsuccessful (more losses). The significant, positive correlation (*r* = 0.202, *p* = 0.036) shows that slowing down decision-making following losses relative to wins increases the likelihood of future successful performance. (**b**) Scatterplot depicting the significant, positive correlation (*r* = 0.295, *p* = 0.002) between the degree of *post-error slowing* and individual expressions of *win-stay* behaviour. (**c**) Scatterplot depicting the lack of a significant correlation (*r* =  − 0.103, *p* = 0.287) between the degree of *post-error slowing* and individual expressions of *lose-shift* behaviour. Across (**a**–**c**), filled-in circles represent participants from Experiment 2 (*n* = 36) and unfilled circles represent individual participants from Experiment 3 (*n* = 36).

A two-way repeated-measures ANOVA was carried out on trial *n* + 1 median RTs using credit type (*no, variable, fixed*) and outcome at trial *n* (*win, lose, draw*; see Fig. 1b, Table 3). The single response selection RT in the *no credit* condition was compared to the first (credit input) response RT in the *fixed credit* condition, and the response selection RT in the *variable credit* condition. Group average data are shown in Fig. 1b and individual data are shown in Supplementary Fig. C2. Analyses revealed a significant main effect of outcome [F(2,70) = 14.09, MSE = 23,832, *p* < 0.001, ƞ_p_^2^ = 0.287] in the absence of a significant main effect of credit type [F(2,70) = 0.07, MSE = 75,370, *p* = 0.930, ƞ_p_^2^ = 0.002] and interaction [F(4,140) = 2.10, MSE = 12,361, p = 0.085, ƞ_p_^2^ = 0.056]. Tukey’s HSD test (*p* < 0.05) revealed both losses (457 ms) and draws (438 ms) yielded significantly faster RTs than wins (543 ms; see Fig. 2).

**Table 3:** Reaction times for the three outcomes (win, lose, draw) as a function of credit (no, variable, fixed) across Experiments 1–3.

	Experiment 1 (unexploitable)			Experiment 2 (exploiting)			Experiment 3 (exploitable)		
**Without outlier removal**									
No	559 (57)	448 (40)	418 (35)	615 (48)	503 (47)	477 (48)	508 (38)	467 (39)	417 (28)
Variable	523 (37)	462 (32)	478 (30)	645 (66)	525 (43)	535 (39)	571 (55)	498 (36)	461 (32)
Fixed	547 (60)	461 (44)	419 (33)	596 (43)	504 (32)	508 (33)	612 (56)	572 (57)	496 (40)

	Win	Lose	Draw	Win	Lose	Draw	Win	Lose	Draw
**With outlier removal**									
No	427 (30)	373 (32)	341 (26)	514 (38)	400 (33)	393 (30)	453 (31)	413 (32)	378 (22)
Variable	401 (37)	355 (32)	341 (39)	492 (48)	459 (47)	462 (42)	482 (34)	445 (29)	414 (31)
Fixed	444 (25)	403 (21)	423 (24)	536 (45)	463 (35)	479 (41)	530 (43)	505 (39)	446 (32)

Standard error in parenthesis.

To allay concerns regarding RT outliers, and to maintain consistency with previous protocols in our laboratory, participants were rejected as a result of their average median RT being at least twice as large as the group average median RT (c.f.^5,10^) within any ANOVA cell, resulting in a reduced sample of 26 in Experiment 1. An identical two-way repeated-measures ANOVA replicated the full sample data: a significant main effect of outcome [F(2,50) = 15.01, MSE = 4651, *p* < 0.001, ƞ_p_^2^ = 0.375], in the absence of a significant main effect of credit type [F(2,50) = 2.15, MSE = 32,678, *p* = 0.127, ƞ_p_^2^ = 0.079] or interaction [F(4,100) = 1.60, MSE = 4898, *p* = 0.182, ƞ_p_^2^ = 0.060]. Again, Tukey’s HSD test (*p* < 0.05) revealed both losses (377 ms) and draws (369 ms) yielded subsequently faster RTs than wins (424 ms).

#### Credit selection

A final set of data unique to the *variable credit* condition was the distribution of credits as a function of outcome (*win, lose, draw;* see Table 4). A one-way repeated-measures ANOVA failed to show significance: F(2,70) = 0.57, MSE = 0.08, p = 0.568, ƞ_p_^2^ = 0.016.

**Table 4:** Distribution of credits entered as a function of preceding outcome (win, lose, draw) across Experiments 1–3.

	Start	Win	Lose	Draw
Experiment 1 (unexploitable)	0.168 (0.044)	0.305 (0.048)	0.237 (0.039)	0.290 (0.037)
Experiment 2 (exploiting)	0.148 (0.051)	0.258 (0.035)	0.359 (0.046)	0.235 (0.030)
Experiment 3 (exploitable)	0.110 (0.035)	0.371 (0.044)	0.280 (0.029)	0.239 (0.032)

Standard error in parenthesis.

### Discussion

The data from Experiment 1 replicated a number of key findings related to quality and speed of contiguous decision-making in a competitive environment. Specifically, high-quality, mixed-strategy (MS) behaviour was more likely following positive outcomes. In other words, following wins, participants stayed with their original response approximately 1/3 of the time and changed to one of two new responses approximately 2/3 of the time. This was in contrast to performance following negative outcomes (specifically, losses), which were characterized by increases in shift behaviour beyond that predicted by MS^3,5^. Moreover, decision-times following negative outcomes were faster than decision-times following positive outcomes, consistent with other unexploitable competitive contexts^43^. Therefore, Experiment 1 provides support for the connection between the valence of the previous competitive encounter and the speed and quality of the next encounter: a negative outcome speeds subsequent decision-making and leads to an overuse of shift behaviour. This highlights the resilience of *lose-shift* behaviour despite its sub-optimality^36,37^. These data were also consistent across *no*, *fixed* and *variable credit* conditions. That is, the addition of an extra response per trial (approximately 400 ms) in the *fixed credit* condition did not change the distribution of participant responding. There was also no evidence that the voluntary decision to slow down the cycle of play via *variable credit* changed responding relative to the *no credit* condition.

One reason why there was no effect of the credit systems in Experiment 1 was that the participants were in no danger of being exploited. Lack of exploitation may also have been the reason why deviations from MS were observed (although does not help to explain why there was over-use of shift behaviour following negative outcomes but *not* over-use of stay behaviour following wins). Slower, improved and/or better-managed decision-making may be observed when there are clearer threats of exploitation. This is evidenced in certain primate data: when a computerized opponent played according to MS, primates were observed to overplay certain responses, but when the opponent began to exploit response selection the primate’s strategy changed to reflect an appreciation of the opponent’s last play^50,51^; see also^52^.

Therefore, Experiment 2 was carried out where the opponent was now designed to take advantage of any item biases expressed by the participant (i.e., *exploiting*). Changing from an *unexploiting* (Experiment 1) to *exploiting* (Experiment 2) opponent should demand more regulation and control of decision-making. By highlighting competitive threat, participants may utilize the *fixed credit* and/or *variable credit* conditions more, in order to slow down their cycle of play and increase in decision-making quality. Specifically, there should be a reduction in shift behaviour as a function of negative outcomes from Experiment 1, and an increased likelihood that participants would play more in accordance with MS. Given that the task in Experiment 2 was to minimize the number of losses rather than maximize the number of wins, it was predicted that—as in Experiment 1—performance should again be characterized by post-error speeding.

## Experiment 2

Experiment 2 was identical to Experiment 1, apart from the change in opponency to *exploiting*. Item biases are a recurring observation in the RPS literature, with Rock currently enjoys a slight over-popularity in empirical studies of the game^3,6,31,53–55^ (although see^56^ for Scissors bias). Experiment 2 was designed to take advantage of *any* idiosyncratic item bias that participants might express throughout the course of the game. The potential exploitation of the participant was made possible by the computer creating a response matrix inverse to the participant’s response selections every 6 trials. For example, if on the first 6 trials the participant selected 4 Rock, 1 Paper and 1 Scissors response, the computer would play for the next 6 trials 4 Paper, 1 Scissors and 1 Rock response (in a random order). Apart from the first six trials where the opponent plays according to MS (2 responses per item), the opponent could exploit any item bias(es) exhibited by the participant on the remaining 84 trials. All other parameters and all statistical analyses were identical to Experiment 1. Two individuals were replaced due the recording of only 89 credits in the variable credit condition. Of the final sample of 36 participants, 3 declined to provide demographic information. Of the remaining sample of 33 individuals, 26 were female and 28 were right-handed (mean age = 20.52, *stdev* = 2.73).

### Results

#### Item and outcome biases

Rock selection did not significantly vary as a function of *no*, *variable* and *fixed* credit conditions [F(2,70) = 0.93, MSE = 0.002, *p* = 0.398, ƞ_p_^2^ = 0.026] nor did the proportion of Rock selection significantly differ from the expected value of 33.3% as assessed by a one-sampled t-test: *t*[35] = 1.17, *p* = 0.252. Similarly, the proportion of win outcomes did not significantly vary as a function of credit conditions [F(2,70) = 1.93, MSE = 0.002, *p* = 0.152, ƞ_p_^2^ = 0.052] nor did win significantly differ from the expected value of 33.3% as assessed by a one-sampled t-test: *t*[35] = 1.17, *p* = 0.252. Table 1 provides descriptive statistics regarding items and outcomes.

For Experiment 2, the arc-sine transformed distribution of *Rock* (34.13%), *Paper* (34.32%), *Scissors* (31.55%) responses significantly varied: [F(2,70) = 3.75, MSE = 0.008, *p* = 0.028, ƞ_p_^2^ = 0.097] but did not interact with condition (*no*, *variable*, *fixed*): [F(4,140) = 0.59, MSE = 0.003, *p* = 0.672, ƞ_p_^2^ = 0.017], where Paper was overplayed relative to Scissors. Arc-sine proportions of *win* (32.59%), *lose* (33.41%) and *draw* (34.00%) outcomes as a function of condition produced no significant main effect of condition: [F(2,70) = 1.91, MSE = 0.003, *p* = 0.156, ƞ_p_^2^ = 0.052], nor an interaction: [F(4,140) = 1.35, MSE = 0.005, *p* = 0.253, ƞ_p_^2^ = 0.037].

#### Reinforcement learning biases

*Win-stay* behaviour did not alter as a function of condition: [F(2,70) = 1.41, MSE = 0.030, *p* = 0.250, ƞ_p_^2^ = 0.038] and the observed average value of 30.15% did not differ significantly from the expected value of 33.33% (*t*[35] =  − 1.15, *p* = 0.257). *Lose-shift* behaviour did not alter as a function of condition: [F(2,70) = 1.34, MSE = 0.009, *p* = 0.268, ƞ_p_^2^ = 0.037] but the observed average value of 80.18% did differ significantly from the expected value of 66.66% (*t*[35] = 9.11, *p* < 0.001). *Draw-shift* behaviour did not alter as a function of condition: [F(2,70) = 0.43, MSE = 0.014, *p* = 0.653, ƞ_p_^2^ = 0.012] but the observed average value of 73.70% did differ significantly from the expected value of 66.66% (*t*[35] = 4.47, *p* < 0.001). The degree of shift behaviour following loss was greater than that following draw (*t*[35] = 3.46, *p* = 0.001; two-tailed; see Figure and 1a and Table 2).

#### Reaction times

A two-way repeated-measures ANOVA on trial *n* + 1 median RTs using credit type (*no, variable, fixed*) and outcome at trial *n* (*win, lose, draw*; see Fig. 1b and Table 3) revealed a significant main effect of outcome [F(2,70) = 15.84, MSE = 27,581, *p* < 0.001, ƞ_p_^2^ = 0.312] in the absence of a significant main effect of credit type [F(2,70) = 0.43, MSE = 100,698, *p* = 0.650, ƞ_p_^2^ = 0.012] and interaction [F(4,140) = 0.42, MSE = 17,775, p = 0.793, ƞ_p_^2^ = 0.011]. Tukey’s HSD test (*p* < 0.05) revealed both losses (511 ms) and draws (507 ms) yielded significantly faster RTs than wins (619 ms).

Following the removal of 10 individuals for Experiment 2 (see Experiment 1 for details), the significant main effect of outcome was replicated [F(2,50) = 12.23, MSE = 10,949, *p* < 0.001, ƞ_p_^2^ = 0.329] in the absence of a significant main effect of credit type [F(2,50) = 0.96, MSE = 67,351, *p* = 0.391, ƞ_p_^2^ = 0.037] and interaction [F(4,100) = 2.36, MSE = 7214, p = 0.059, ƞ_p_^2^ = 0.086]. Tukey’s HSD test (*p* < 0.05) revealed both losses (441 ms) and draws (445 ms) yielded significantly faster RTs than wins (514 ms).

#### Credit selection

A one-way repeated-measures ANOVA failed to show significance in credit distribution as a function of outcome in the *variable* condition (see Table 4): F(2,70) = 2.53, MSE = 0.06, p = 0.087, ƞ_p_^2^ = 0.067.

### Discussion

Experiment 2 tested the idea that the failure to extend or truncate decision-making times via the use of credit systems was due to there being no negative consequences for deviation from optimal performance (*lose-shift*). If an opponent exploited these deviations, then behaviour should more closely align with MS, especially when given more (*fixed credit*, *variable credit*) rather than less (*no credit*) time to make decisions. However, at a group level, participants fared no worse against an *exploiting* (Experiment 2) versus *unexploitable* (Experiment 1) opponent as *lose* rates were not significantly different (33.41% vs. 33.89%, respectively; between-participants t-test: *t*[70] = 0.82, *p* = 0.410). Furthermore, the variance of lose rates was not significantly smaller in Experiment 2 relative to Experiment 1 (Levene’s test: *t*[70] = 0.85, p = 0.360)—something that might have been predicted if participants were more likely to operate under mixed-strategy to avoid exploitation in Experiment 2 but not Experiment 1. A final possibility is that any *exploiting* opponent designed with a static rule of course could be reconfigured to become an *exploitable* opponent. The idea that there is a variety of individual experiences against non-mixed-strategy opponents will be revisited.

Nevertheless, Experiment 2 replicated Experiment 1 in two important ways. First, the data continued to show the increased use of *shift* behaviour following negative outcomes over that predicted by mixed-strategy. The idea that *lose-shift* behaviour reliably manifests itself against putatively *unexploitable* (Experiment 1) and *exploiting* (Experiment 2) opponents suggests something of the immutability of this particularly reinforcement learning rule, relative to the flexibility observed in the expression of *win-stay* behaviour. This observation is consistent with previous human data where *win-stay* but not *lose-shift* behaviour modulated as a function of outcome value^5^, electrophysiological work where feedback-related negative (FRN) to wins but not losses modulate as a function of frequency^57^, and, also animal work where *lose-shift* is seen as a more hard-wired reflex^35,36^. These ideas also align with the principle of loss aversion^58^ (although see^59^), loss attention whereby negative outcomes decrease inertia^13^, and evolutionary accounts where avoiding the damage following losing is more important that reaping the benefits following success^34,35^. Second, the RT data suggests that part of the reason for this sub-optimal behaviour may be the self-imposed reduction in time allocated to decisions following negative outcomes (i.e., *post-error speeding*). In a final attempt to explore the relationship between the quality, speed and control of competitive decision-making in Experiment 3, we exposed participants to an *exploitable* opponent.

## Experiment 3

Previous work suggests that the development of a mental model leading to the successful exploitation of an opponent can radically change competitive performance. For example, *post-error speeding* becomes *post-error slowing* during successful exploitation, with the degree of slowing predicted by the degree of exploitation^10^. Therefore, it is possible that the sense of environmental control established during successful exploitation will also translate to an increased utility for varying decision-making times via credit systems.

In terms of the specific *exploitable* rule used in Experiment 3^60^, if a computer opponent plays one item more often than another (e.g., Rock) then human participants will learn to play the appropriate counter-item with increased frequency (e.g., Paper; see also *secondary salience*^61^). Therefore, opponents with item biases should lead to increases in both *win-stay* and *lose-shift* participant behaviours. This is because increasing the frequency of item repetition for an opponent should similarly reinforce the repetition of a participant’s item following wins and also reinforce the change of a participant’s item following losses. By observing the degree of change across *win-stay* and *lose-shift* proportions, *exploitable* opponents serve as a final test of flexibility between these reinforcement learning heuristics.

Experiment 3 was identical to both Experiment 2, apart from the change in opponency to *exploitable*. Here, opponents in each of the three conditions (*no*, *variable* and *fixed*) were given an item bias of 51.11%. For example, Rock was played for 46 trials whereas both Paper and Scissors were played for 22 trials each, in a random order. The assignment of item bias (R, P, S) to condition was counterbalanced, as was the order of conditions. All other parameters and all statistical analyses were identical to Experiments 1 and 2. Two individuals were replaced due the recording of only 89 credits in the variable credit condition. Of the final sample of 36 participants, 1 declined to provide demographic information. Of the remaining sample of 35 individuals, 23 were female and 32 were right-handed (mean age = 21.46, *stde*v = 5.05).

### Results

#### Item and outcome biases

Rock selection did not significantly vary as a function of *no*, *variable* and *fixed* credit conditions [F(2,70) = 0.37, MSE = 0.012, *p* = 0.691, ƞ_p_^2^ = 0.010] nor did the proportion of Rock selection significantly differ from the expected value of 33.3% as assessed by a one-sampled t-test: *t*[35] = 0.96, *p* = 0.343. However, wins did significantly vary as a function of credit conditions [F(2,70) = 4.80, MSE = 0.003, *p* = 0.011, ƞ_p_^2^ = 0.121], and were increased in the *variable credit* condition relative to the *fixed credit* condition (37.28% vs. 33.49%; see Table 1)*.* Global win rates were also significantly higher than the expected value of 33.3% as assessed by a one-sampled t-test: *t*[35] = 3.26, *p* = 0.003, and were significantly greater than the win rates experienced against an exploiting opponent in Experiment 2: between-participants t-test: *t*[70] = 3.76, p < 0.001.

For Experiment 3, the arc-sine transformed distribution of *Rock* (34.88%), *Paper* (32.84%), *Scissors* (32.28%) responses did not vary: [F(2,70) = 0.56, MSE = 0.042, *p* = 0.576, ƞ_p_^2^ = 0.016] nor interact with condition (*no*, *variable*, *fixed*): [F(4,140) = 0.65, MSE = 0.028, *p* = 0.628, ƞ_p_^2^ = 0.018]. Arc-sine proportions of *win*, *lose* and *draw* outcomes produced a significant main effect: [F(2,70) = 6.08, MSE = 0.006, *p* = 0.004, ƞ_p_^2^ = 0.148] as well as an interaction: [F(4,140) = 2.70, MSE = 0.004, *p* = 0.033, ƞ_p_^2^ = 0.072]. The increase in wins relative to losses and draws expected as a result of the opponent being exploitable was only significant in the *variable credit* condition (see Table 1).

A small but significant item bias for Rock was revealed across Experiments 1–3, with the observed value of 34.73% different from the expected value of 33.3%: *t*[107] = 2.07, *p* = 0.040. This is consistent with previous work^3,6,31,53–55^. A binomial test was also carried out for each individual under the null hypothesis that their average proportion of Rock was 33.3% and the null could be rejected (α = 0.050) for 100 out of 108 individuals, of whom 58 showed Rock selection *above* the value expected by mixed strategy. The most parsimonious explanation for this effect is a primary effect^62^, akin to the over selection of Heads in the two-response game Matching Pennies, where participants have a tendency to select the first item. This 58% is similar in magnitude to other ‘majorities’ reported in decision-making work (e.g., the 55% of individuals who demonstrate more environmental sampling in loss relative to gain experimental contexts^16^, p. 338).

#### Reinforcement learning biases

*Win-stay* behaviour did not alter as a function of condition: [F(2,70) = 0.99, MSE = 0.021, *p* = 0.376, ƞ_p_^2^ = 0.028] and the observed average value of 43.88% did differ significantly from the expected value of 33.33% (*t*[35] = 3.16, *p* = 0.003). *Lose-shift* behaviour did alter as a function of condition: [F(2,70) = 3.72, MSE = 0.014, *p* = 0.029, ƞ_p_^2^ = 0.096], with *no* credit (77.48%) varying from *fixed* (69.80%) but not *variable* credit (74.31%; Tukey’s HSD, *p* < 0.05). The observed average value for *lose-shift* behaviour (73.86%) significantly differed from the expected value of 66.66% (*t*[35] = 2.55, *p* = 0.015). *Draw-shift* behaviour did not alter as a function of condition: [F(2,70) = 1.59, MSE = 0.018, *p* = 0.212, ƞ_p_^2^ = 0.043] and the observed average value of 66.09% did not differ significantly from the expected value of 66.66% (*t*[35] = 0.17, *p* = 0.864). The degree of shift behaviour following loss was greater than that following draw (*t*[35] = 3.46, *p* = 0.001; two-tailed; see Fig. 1, Table 2).

#### Reaction times

A two-way repeated-measures ANOVA on trial *n* + 1 median RTs using credit type (*no, variable, fixed*) and outcome at trial *n* (*win, lose, draw*; see Fig. 2, Table 3) revealed a significant main effect of outcome [F(2,70) = 15.90, MSE = 19,083, *p* < 0.001, ƞ_p_^2^ = 0.308] in the absence of a significant main effect of credit type [F(2,70) = 1.89, MSE = 130,885, *p* = 0.158, ƞ_p_^2^ = 0.051] and interaction [F(4,140) = 0.34, MSE = 16,148, p = 0.850, ƞ_p_^2^ = 0.010]. Tukey’s HSD test (*p* < 0.05) revealed wins (563 ms), losses (512 ms) and draws (458 ms) were all significantly different from one another.

Following the removal of 7 individuals for Experiment 3, a two-way repeated-measures ANOVA on trial *n* + 1 median RTs using credit type (*no, variable, fixed*) and outcome at trial *n* (*win, lose, draw*; see Fig. 2, Table 3) revealed a significant main effect of outcome [F(2,56) = 13.63, MSE = 9018, *p* < 0.001, ƞ_p_^2^ = 0.328] in the absence of a significant main effect of credit type [F(2,56) = 2.09, MSE = 65,379, *p* = 0.133, ƞ_p_^2^ = 0.069] and interaction [F(4,112) = 0.29, MSE = 6018, p = 0.886, ƞ_p_^2^ = 0.010]. RTs for losses (454 ms) and draws (413 ms) were faster RTs than wins (488 ms), although only the difference between draws and wins was significant (Tukey’s HSD, *p* < 0.05).

#### Credit selection

A one-way repeated-measures ANOVA on credit distribution was significant: F(2,70) = 3.51, MSE = 0.05, p = 0.035, ƞ_p_^2^ = 0.091, showing that significantly more credits were entered following wins relative to draws (see Table 4).

## Cross-experiment comparison

A number of central conclusions can be drawn by summarizing the data across Experiments 1–3. First, the data reliably show that reaction times following losses were faster (or more ‘impulsive’^43^) than reaction times following wins (see also^6^, Experiment 1). Such *post-error speeding* has previously been conceptualized as a self-imposed reduction in time allocated to decisions following losses, with the individual aiming to exiting the failure state as quickly as possible (contra^13^). However, this raises the concern that the less time one thinks about one’s next decision, the more likely it is to be sub-optimal, giving rise to cycles of poor performance. To investigate these ideas further, RT differences between losses and wins (collapsed across credit condition) were calculated on an individual basis for the two experiments in which a model of opponent performance could be learnt (Experiments 2 and 3; *n* = 72), and, compared with the difference between win and loss rates experienced by the same participant (following^10^). Figure 2a depicts a significant, positive correlation between the degree of success exhibited by the participants (i.e., more wins) and the degree to which decisions following losses were slower than decisions following wins (i.e., *post-error slowing*; *r* = 0.202, *p* = 0.036). Thus, slowing down decision-making following losses increases the likelihood of future successful performance.

Two further correlations were examined in an attempt to pinpoint which reinforcement learning mechanism might be more sensitive to promotion following extra decision-making time. A significant, positive correlation between post-error slowing and *win-stay* rates (*r* = 0.295, *p* = 0.002; Fig. 2b) was contrasted with a non-significant, negative correlation between post-error slowing and *lose-shift* rates (*r* =  − 0.103, *p* = 0.287; Fig. 2c). Therefore, reduced impulsivity exhibited by participants following loss was also linked to the ability to re-initiate successful *win-stay* but not *lose-shift* strategies.

This ease with which *win-stay* behaviour can be initiated, relative to the inflexibility of *lose-shift* behaviour, was further reinforced by also looking across studies in another way. The average proportion of *win-stay* behaviour (36.67%, 30.15%, 43.88%, respectively) was compared to the average proportion of *lose-shift* behaviour (77.23%, 80.18%, 73.86%, respectively) across Experiments 1–3 in a two-way, mixed ANOVA (behaviour as a within-participants factor, and, experiment as a between-participants factor). There was no main effect of experiment: F(2,105) = 1.27, MSE = 0.02, *p* = 0.287, ƞ_p_^2^ = 0.024, but there was a main effect of behaviour: F(1,105) = 293.78, MSE = 0.03, *p* < 0.001, ƞ_p_^2^ = 0.730, as well as an experiment × behaviour interaction: F(2,105) = 5.89, MSE = 0.03, *p* = 0.004, ƞ_p_^2^ = 0.101. The only significant difference to arise from the current data set was the difference in *win-stay* behaviour between the *exploiting* opponent (Experiment 2) and the *exploitable* opponent (Experiment 3; Tukey’s *p* < 0.05). This is consistent with previous data where *win-stay* rates modulate to a greater degree than *lose-shift* rates, highlighting that the former is under cognitive control whereas the latter retains more of a reflexive quality. This is, of course, not to suggest that *lose-shift* behaviour could not be attenuated using substantially longer delays between decisions (c.f, 1, 6.5 and 12 s lags used^45^), simply to say it is easier to stay following wins than it is to shift following loss.

## General discussion

The main goal of this paper was to specifically test the relationship between decision-making speed and quality, with the hypothesis that slowing down decision-making following losses increases the likelihood of future successful performance in cases where a successful model of opponent domination can be implemented. The data confirm that self-imposed reductions in processing time following losses (*post-error speeding*) are causal factors in determining poorer-quality behaviour (see Fig. 2). Specifically, the data also provide evidence that *win-stay* (rather than *lose-shift* mechanisms) might be more sensitive to promotion following extra decision-making time.

Second, the data reinforce the inflexibility of *lose-shift* as a decision-making heuristic in competitive contexts. However, it is important to address the idea that the potency of the *lose-shift* heuristic may in part be due to the weakness of the opponent manipulation across Experiments 1–3. Relative to the *unexploitable* opponent in Experiment 1, where we expected average win rates to be around 1/3 (32.93%) as a result of the use of MS, we did not see a reduction in win rate in Experiment 2 (32.59%) when the opponent was designed to take advantage of any transitory idiosyncratic item bias that participants might have (*exploiting*). Moreover, while significantly different from Experiment 2, the average win rate against *exploitable* opponents in Experiment 3 (35.30%) was not comparable to the degree of success observed in previous studies against *exploitable* opponents (c.f.^10^ where participants achieved 18–24% differential between their win and lose rate). It is clear that more extreme expressions of *exploiting* and *exploitable* opponents should be used in future research. However, since there can be no guarantee that participant will offer themselves up to exploitation—nor take advantage of opponent who are themselves exploitable^63^—an alternative approach may be to design *exploitable* and *exploiting* conditions with fixed rather than variable win rates^57,64–66^. For example^67^ created an 80–20% differential between win and lose trials that could be used to recreate exploitable and exploiting opponents, respectively. One critical issue however with the use of fixed outcomes is that there is no consistency either within or between participants in the behaviour that will ultimately be reinforced or punished. This may have large scale consequences for how behaviour is perceived and the degree to which participants believe success and failure is under their control.

Finally, the data provide future directions for understanding how the use of a variable ‘credit’ (or ‘token’) system may enhance the perceived control participants have against exploitable opponents. Specifically, win rates against *exploitabl*e opponents (Experiment 3) were enhanced in the *variable credit* condition, and, participants also inserted more credits following wins in the *variable credit* condition. This interaction between *variable credit* and *exploitable* opponents may reflect an increased sense of control^18,20^, as a result of the successful implementation of a mental model of the competitive environment. Relative to *unexploitable* or (*prima facie*) *exploiting* opponents, *exploitable* opponents offer a clear opportunity for strategic learning, where success is clearly indexed by an increase in win rate. Similarly, performance during the *variable credit* condition was also characterized by an increased opportunity for environmental control: the game slows and speeds according to the distribution of credits dictated by the participant. Importantly, the observation that more credits were inserted following win trials against exploitable opponents suggests that participants were initiating their own form of *post-reinforcement pause*^8^: increasing the time allocated to decision-making on the next round, thereby increasing their chances of consecutive success.

In future work, it will be important to disentangle two features of any putative credit system: temporal lag and response interruption^68^. Relative to the *no credit* conditions, both *fixed* and *variable credit* conditions extended the time between trials (temporal lag) as a result of requiring participants to switch from their RPS task to a credit entering task (see^69^, for a review on the extensive task-switching literature). Therefore, any potential costs or benefits accrued from credits systems could be due to (a) providing individual with more time to make better decisions, (b) disrupting cyclic or poorer-quality motor patterns associated with response selection, or, (c) a combination of the two. Some of our future research will be focused around using average reaction time derived from a *fixed credit* block as an average delay time around which participants are *only* exposed to temporal lags between trials. This type of manoeuver will help to reveal any effects of temporal delay independently of the contribution of response interruption, in the larger context of dynamic decision-making against numerous styles of opponency.

## Supplementary Information

Supplementary Information 1.

Supplementary Information 2.

Supplementary Information 3.

Supplementary Information 4.
