# Supplementary material for: Variability in competitive decision-making speed and quality against exploiting and exploitative opponents
Source: Sci Rep. 2021 Feb 3;11:2859. doi: 10.1038/s41598-021-82269-2 (PMC7859242; doi:10.1038/s41598-021-82269-2)
Supplement: Supplementary file 4 — Supplementary Information 4. [file 41598_2021_82269_MOESM4_ESM.doc]

Variability in competitive decision-making speed and quality against exploiting and exploitative opponents

Benjamin James Dyson ^1,2,3^

^1^ University of Alberta, Canada

^2^ Ryerson University, Canada

^3^ University of Sussex, UK

Address : Department Of Psychology

P-217 Biological Sciences Building

University of Alberta

Edmonton

AB

T6G 2E9

Canada

E-mail : [bjdyson@ualberta.ca](mailto:bjdyson@ualberta.ca)

***SUPPLEMENTARY INFORMATION A*** *On-screen instructions*

*No Credit*

You are invited to play 90 rounds of Rock Paper Scissors

At each trial, select one of three options using the keypad:

4 ROCK 5 PAPER 6 SCISSORS

You will then see a picture of a blue glove and a white glove playing Rock, Paper, Scissors.

Your OPPONENT is the BLUE glove and YOU are the WHITE glove.

The picture will depict your response and your opponent’s response.

The computer will then tell you whether you WON, LOST or DREW that trial.

--------------------------------------------------------------------------------

You will get +1 point for a win, -1 for a loss and 0 for a draw.

--------------------------------------------------------------------------------

Your current score can always be seen at the bottom of the screen. Try and accumulate the highest score possible across the trials!

Your opponents may play in different ways and may use different strategies to try and win.

A reminder on the rules of Rock, Paper, Scissors:

ROCK beats SCISSORS

PAPER beats ROCK

SCISSORS beats PAPER

Please do not spend too long thinking about your answer as we are interested in your intuitive responses.

In this condition, each trial only requires the input of your R or P or S response.

Press 0 to start.

*Fixed Credit*

You are invited to play 90 rounds of Rock Paper Scissors

At each trial, select one of three options using the keypad:

4 ROCK 5 PAPER 6 SCISSORS

You will then see a picture of a blue glove and a white glove playing Rock, Paper, Scissors.

Your OPPONENT is the BLUE glove and YOU are the WHITE glove.

The picture will depict your response and your opponent’s response.

The computer will then tell you whether you WON, LOST or DREW that trial.

--------------------------------------------------------------------------------

You will get +1 point for a win, -1 for a loss and 0 for a draw.

--------------------------------------------------------------------------------

Your current score can always be seen at the bottom of the screen. Try and accumulate the highest score possible across the trials!

Your opponents may play in different ways and may use different strategies to try and win.

A reminder on the rules of Rock, Paper, Scissors:

ROCK beats SCISSORS

PAPER beats ROCK

SCISSORS beats PAPER

Please do not spend too long thinking about your answer as we are interested in your intuitive responses.

In this condition, each trial requires you to insert 1 credit (by pressing '0') and then the input of your R or P or S response.

Press 0 to start.

*Variable Credit*

You are invited to play 90 rounds of Rock Paper Scissors

At each trial, select one of three options using the keypad:

4 ROCK 5 PAPER 6 SCISSORS

You will then see a picture of a blue glove and a white glove playing Rock, Paper, Scissors.

Your OPPONENT is the BLUE glove and YOU are the WHITE glove.

The picture will depict your response and your opponent’s response.

The computer will then tell you whether you WON, LOST or DREW that trial.

--------------------------------------------------------------------------------

You will get +1 point for a win, -1 for a loss and 0 for a draw.

--------------------------------------------------------------------------------

Your current score can always be seen at the bottom of the screen. Try and accumulate the highest score possible across the trials!

Your opponents may play in different ways and may use different strategies to try and win.

A reminder on the rules of Rock, Paper, Scissors:

ROCK beats SCISSORS

PAPER beats ROCK

SCISSORS beats PAPER

Please do not spend too long thinking about your answer as we are interested in your intuitive responses.

In this condition, you can store as many credits (by pressing '0') as you wish and then only input your R or P or S response.

Once your stored credits run out, you will have to input more. Once your 90 credits run out, the block will end.

Press 0 to start.

***SUPPLEMENTARY INFORMATION B*** *On-screen displays*


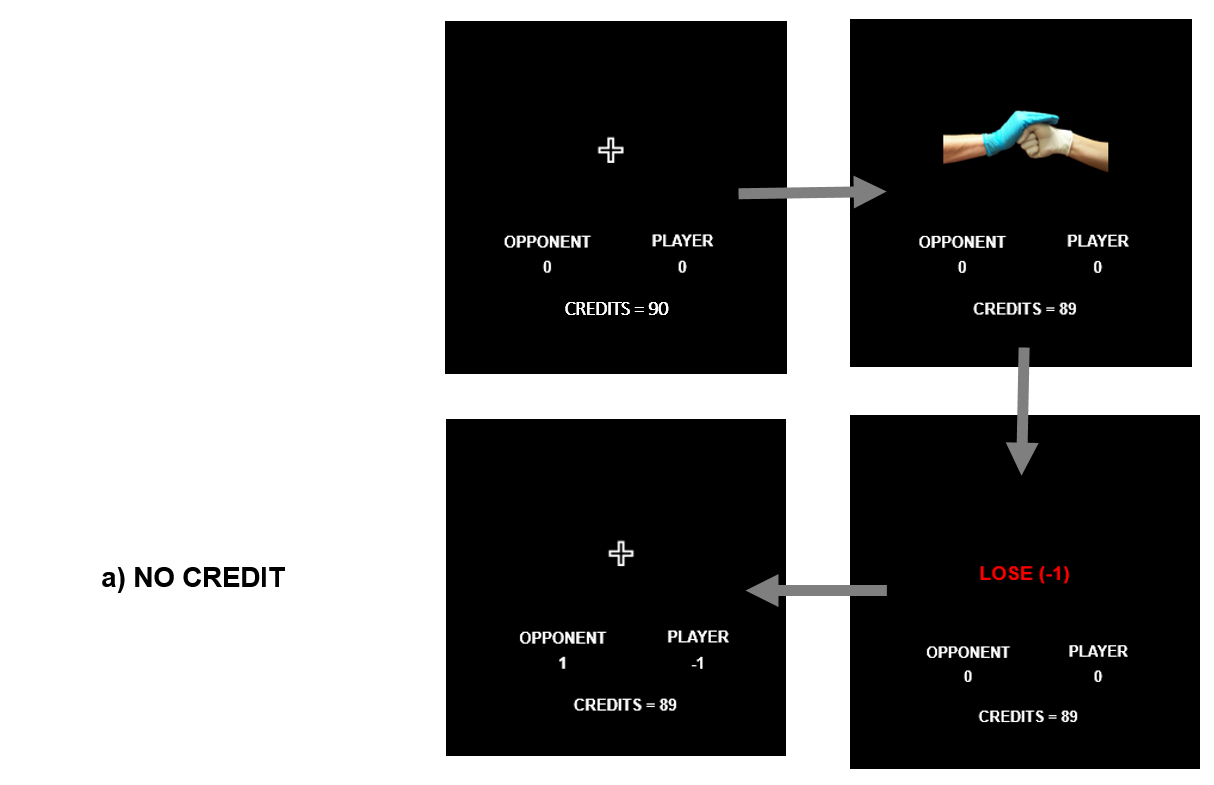
Figure depicts the start screen for a) no credit, b) fixed credit and c) variable credit condition, and depicts the main trial events for the variable credit condition*.*

*
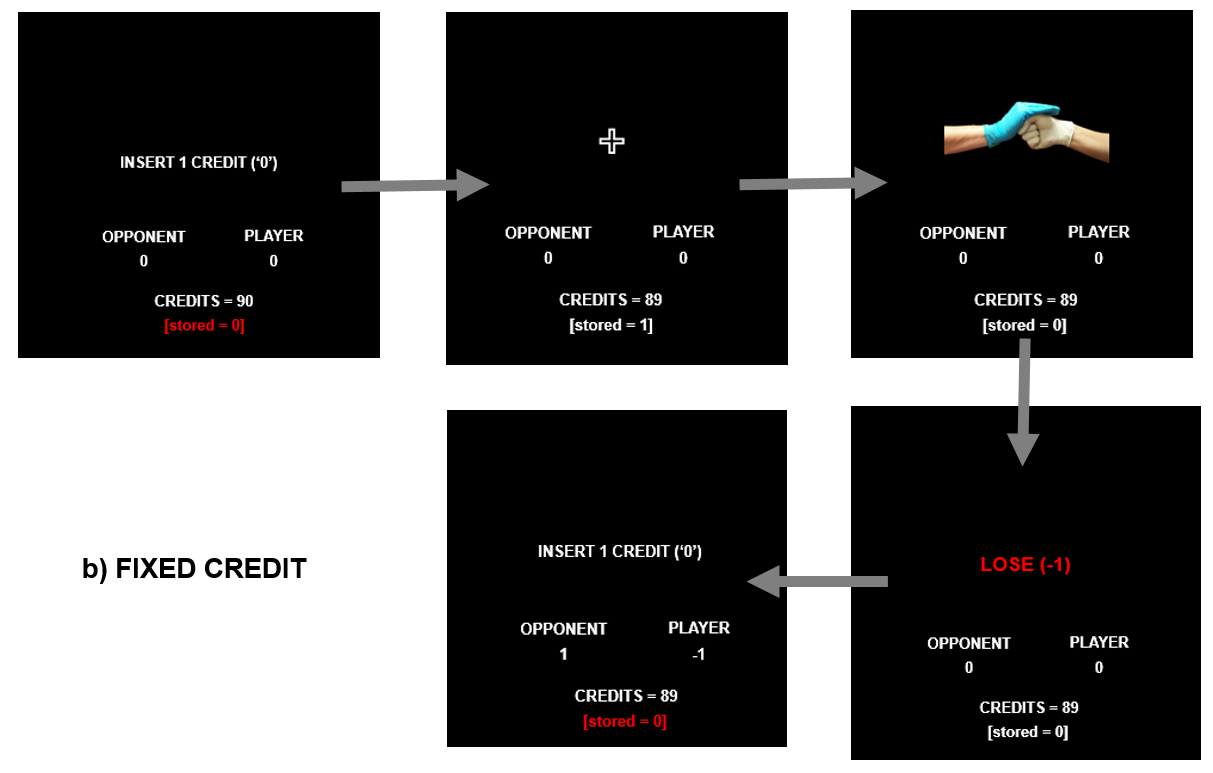
*

*
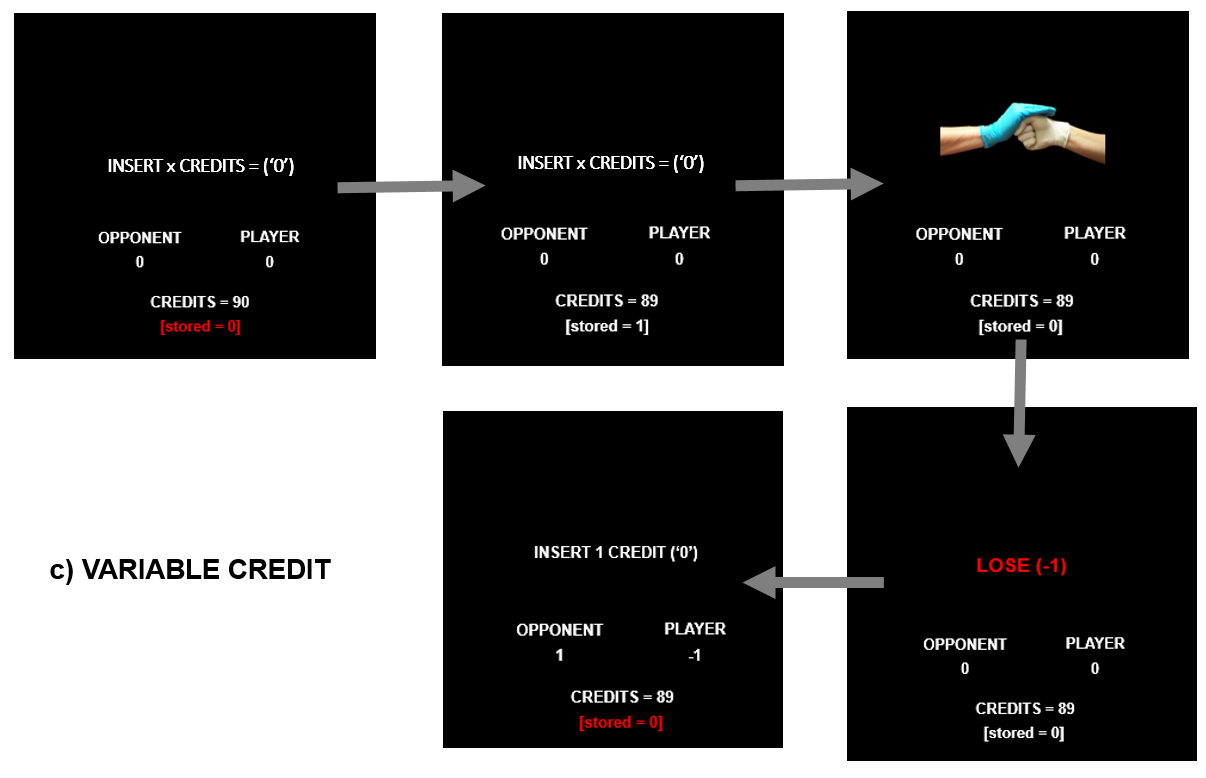
*

***SUPPLEMENTARY INFORMATION C1 & C2***

*Figure Captions*

*Figure C1*  Graph showing proportion of reinforcement learning biases (*win-stay*, *lose-shift*, *draw-shift*) under conditions of *no, variable* and *fixed* credit across three different opponent styles (Experiment 1 = unexploitable, Experiment 2 = exploiting, Experiments 3 = exploitable). Dotted lines represent expected values of 33.3% in *win-stay*, and, 66.6% in both *lose-shift* and *draw-shift* behaviours. Grey lines represent individual data, black lines represent mean data with error bars indicate +/- 1 standard error.

*Figure C2*  Graph showing reaction times at trial *n+1* separated by outcome at trial *n (win, lose, draw)* under conditions of *no, variable* and *fixed* credit across three different opponent styles (Experiment 1 = unexploitable, Experiment 2 = exploiting, Experiments 3 = exploitable). Grey lines represent individual data, black lines represent mean data with error bars indicate +/- 1 standard error.

*Supplementary Figure C1*

*
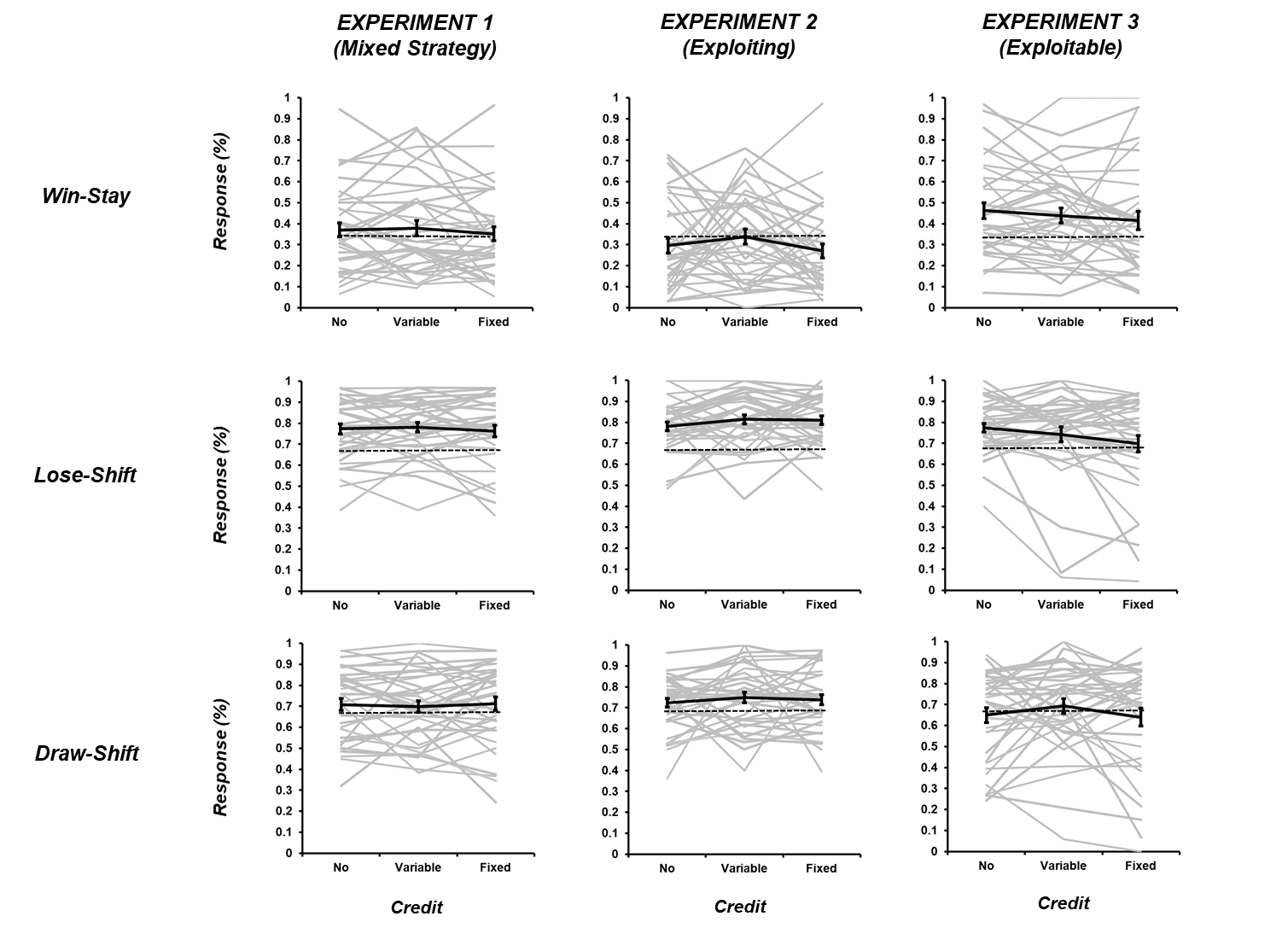
*

*Supplementary Figure C2*

*
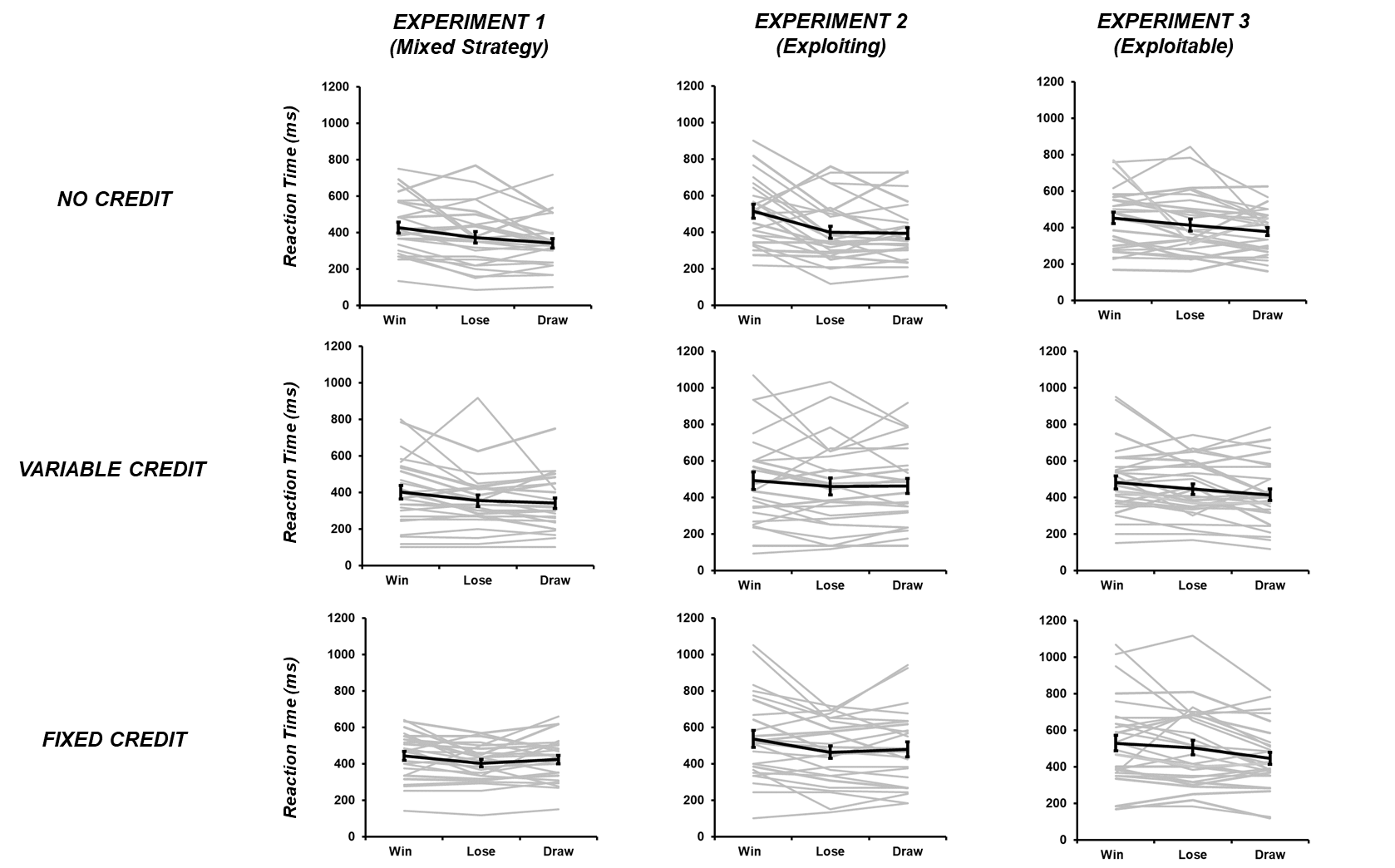
*
